# Lp-PLA2 Inhibition—The Atherosclerosis Panacea?

**DOI:** 10.3390/ph3051360

**Published:** 2010-04-29

**Authors:** Mahir Karakas, Wolfgang Koenig

**Affiliations:** Department of Internal Medicine II-Cardiology, University of Ulm Medical Center, Ulm, Germany; E-Mail: mahir.karakas@uniklinik-ulm.de (M.K.)

**Keywords:** Lp-PLA_2_, inflammation, oxidative processes, atherosclerosis, specific inhibition

## Abstract

Based on the complex pathophysiology of atherosclerosis, a large number of biomarkers that relate to lipids, inflammation, immunity, thrombosis and hemostasis, have been investigated experimentally, in epidemiologic studies and in clinical trials. Interest focuses on their potential role to aid in risk stratification, as possible surrogate markers of atherosclerosis, and potential targets for therapy. More recently, one lipid associated biomarker, lipoprotein-associated phospholipase A2 (Lp-PLA_2_), has gained considerable interest. In addition to a plausible pathophysiological role by generating pro-inflammatory and pro-atherogenic compounds from oxidized LDL in the vessel wall, there is a large, fairly consistent epidemiological database indicating that increased levels of Lp-PLA_2 _ mass or activity are associated with increased risk for cardiovascular outcomes; such data further suggest that it might improve risk stratification. In addition, clinical studies indicate that increased Lp-PLA_2_ levels are associated with endothelial dysfunction. Moreover, it may also serve as an interesting therapeutic target, since a specific inhibitor of the enzyme is available with promising animal data and initial positive data in humans. Recent experimental data from a hyperlipidemic diabetic pig model strongly suggest that increased Lp-PLA_2 _ in the vessel wall is associated with a more vulnerable plaque phenotype which can be modulated by inhibiting Lp-PLA_2_ activity. A biomarker study in more than 1,000 patients with CHD over three months has demonstrated a positive effect on various inflammatory molecules. In addition, an imaging study using IVUS based modalities (greyscale, virtual histology, and palpography) together with a panel of biomarkers (IBIS-2) has been done in more than 300 patients with CHD treated over 12 months and results indicate that the progression of the necrotic core of the plaque can be retarded. Inhibition of the pro-atherogenic and pro-inflammatory effects of Lp-PLA_2_ may therefore contribute to decrease the residual risk in high risk patients already on polypharmacotherapy. This hypothesis is now being tested in two large phase 3 clinical trials. Thus, Lp-PLA_2_ indeed may represent a biomarker and a promising target for intervention.

## 1. Biochemistry and Biology

Based on the complex pathophysiology of atherosclerosis, a large number of biomarkers that relate to lipids, inflammation, immunity, thrombosis and hemostasis, have been investigated experimentally, in epidemiologic studies, and in clinical trials. Interest focuses on their potential role to aid in risk stratification, as possible surrogate markers of atherosclerosis, and potential targets for therapy. More recently, one lipid associated biomarker, lipoprotein-associated phospholipase A_2_ (Lp-PLA_2_), has gained considerable interest.

**Figure 1:**
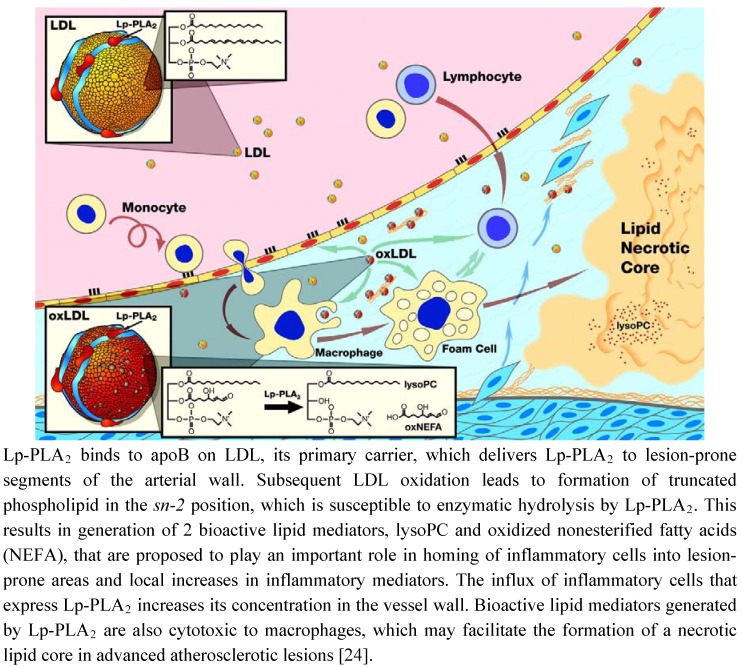
Schematic representation of the proposed pro-atherogenic mechanism of Lp-PLA_2_ in the vessel wall.

Lp-PLA_2_, also known as secretory phospholipase A_2_ group VII (sPLA_2_-VII) and as platelet activating factor acetylhydrolase (PAF-AH), is widely expressed in cells involved in atherosclerosis, such as macrophages, T-cells, lymphocytes, and mast cells [1,2]. It is a calcium-independent serin lipase that hydrolyzes phospholipids at the *sn*-2 position and acts preferentially on water-soluble polar phospholipids, particularly those with oxidatively truncated fatty acids [3,4]. Via a specific protein-protein interaction between the N-terminus of Lp-PLA_2_ and the C-terminus of apolipoprotein B (apoB) two-third of the Lp-PLA_2_ circulates primarily bound to LDL cholesterol while the remaining third is distributed between high-density lipoprotein (HDL) cholesterol and very-low-density lipoproteins (VLDL) [5]. The oxidation of LDL cholesterol within the arterial wall provides the substrate for the hydrolytic action of Lp-PLA_2_ -a short acyl group at the sn-2 position of phospholipids. By cleaving an oxidized phosphatidylcholine component of the lipoprotein particle, Lp-PLA_2_ generates potent proinflammatory and proatherogenic mediators like oxidized nonesterified fatty acids (ox-FA), arachidonic acid and lysophosphatidylcholine (Lyso-PC) [6] (Figure 1). Since inflammation plays a major role at all stages of atherogenesis, from endothelial dysfunction to plaque development and ultimately to plaque rupture, Lp-PLA_2_ may contribute to atherosclerosis by generation of various proinflammatory lipid mediators, including Lyso-PC, ox-FA, and arachidonic acid [7]. Ox-FA´s promote atherosclerosis by directly and indirectly increasing oxidative stress and the presence of oxidized LDL and other lipoproteins in the plasma and arterial walls, thereby initiating fatty streak formation [8]. Cyclooxygenase converts arachidonic acid to inflammatory mediators like thromboxanes and leukotrienes [9,10]. Lyso-PCs act pro-atherogenic in various early steps of atherosclerosis, are expressed by macrophages in human atherosclerotic lesions, and are increased 5-fold in oxidized LDL compared to normal LDL [11,12,13]. In the arterial wall they upregulate adhesive molecules like vascular cell adhesion molecule (VCAM)-1 and intercellular adhesion molecule (ICAM)-1 [14,15]. Furthermore they promote monocyte migration by inducing monocyte chemotactic protein (MCP)-1 and stimulate Interleukin (IL)-1β, IL-6, and tumor necrosis factor (TNF)-α production and scavenger receptor expression in macrophages in a concentration-dependent manner [16,17,18]. Furthermore, Lyso-PCs also upregulate Lp-PLA_2_ activity, resulting in a viscous cycle, thereby pro-inflammatory mediators are becoming increasingly upregulated, contributing to plaque progression and destabilization [19]. 

Although results from the aforementioned experimental studies seem to be fairly consistent, there has been some controversy regarding the biological role of Lp-PLA_2_ in atherosclerosis, since initially, it was thought to be atheroprotective [20,21]. Adenoviral gene transfer of human Lp-PLA_2_ in ApoE-/- mice reduced VLDL-induced *ex vivo* macrophage adhesion and *in vivo* macrophage homing, thereby resulting in reduced atherosclerosis [22]. Furthermore, the pretreatment of an electronegative LDL cholesterol subfraction from hypercholesterolemic human plasma with recombinant Lp-PLA_2_ completely prevented the pro-apoptotic effects of the LDL subfraction on vascular endothelial cells [23].

### 1.1. Pathoanatomical Evidence

In an atherosclerotic diabetes/hypercholesterolemia swine model which develops advanced coronary lesions within six months, expression of 59 genes in the vasculature, related to cholesterol metabolism, inflammation, and insulin signaling pathways were characterized [25]. Inflammatory genes were more markedly upregulated in coronary arteries than in thoracic aortae and carotids. At six months Lp-PLA_2_ gene was significantly upregulated, indicating the potential role that this molecule plays in the development and progression of atherosclerosis. Mannheim *et al*. [26] determined the expression of Lp-PLA_2_ in 167 carotid artery plaques by immunoblotting and immunostaining. Symptomatic carotid artery plaques were characterized by increased levels of Lp-PLA_2_ and its product Lyso-PC in correlation with markers of tissue oxidative stress, inflammation, and instability, strongly supporting a role for Lp-PLA_2_ in the pathophysiology and clinical presentation of cerebrovascular disease. In a recently published study [27] carotid artery plaque expression of Lp-PLA_2_ was quantified in 162 consecutive patients undergoing elective carotid endarterectomy. Follow-up for cardiac death and non-fatal acute myocardial infarction was accomplished over a period of 48 ± 14 months. Carotid plaque Lp-PLA_2_ expression above the median constituted a more than three times higher risk for cardiac events [HR 3.39 (1.13–10.17), P = 0.03]. The relative expression of Lp-PLA_2_ in coronary plaque phenotypes, including unstable lesions, has first been established by Kolodgie *et al*. [28]. They prospectively collected coronary segments (n = 30) from 25 sudden coronary death patients for immunolocalization of Lp-PLA_2_, and showed that Lp-PLA_2_ was strongly expressed within the necrotic core and surrounding macrophages of vulnerable and ruptured plaques, with relatively weak staining in less advanced lesions, suggesting a potential role in promoting plaque instability.

A study of nuclear families attributed 62% variance of Lp-PLA_2_ activity to heritability, although until now few genetic determinants of Lp-PLA_2_ have been identified, and the data for these genetic factors are inconsistent [29]. Most recently, a study in monozygotic and dizygotic twins of Caucasian origin was conducted, to investigate the heritability of plasma levels (mass) and activity of Lp-PLA_2 _[30]. The authors reported that when phenotypic covariance was partitioned into additive genetic effects, environmental effects common to co-twins, and error variance components, a non-negligible component of both Lp-PLA_2_ mass and activity was accounted for by genetic effects, suggesting that both Lp-PLA_2_ activity and mass variance may be genetically determined, although the heritability estimates were only significant for Lp-PLA_2_ activity. Interestingly, these heritability estimates were remarkably similar to the above mentioned estimate variance in US nuclear families. 

## 2. The Epidemiologic Evidence

**Figure 2:**
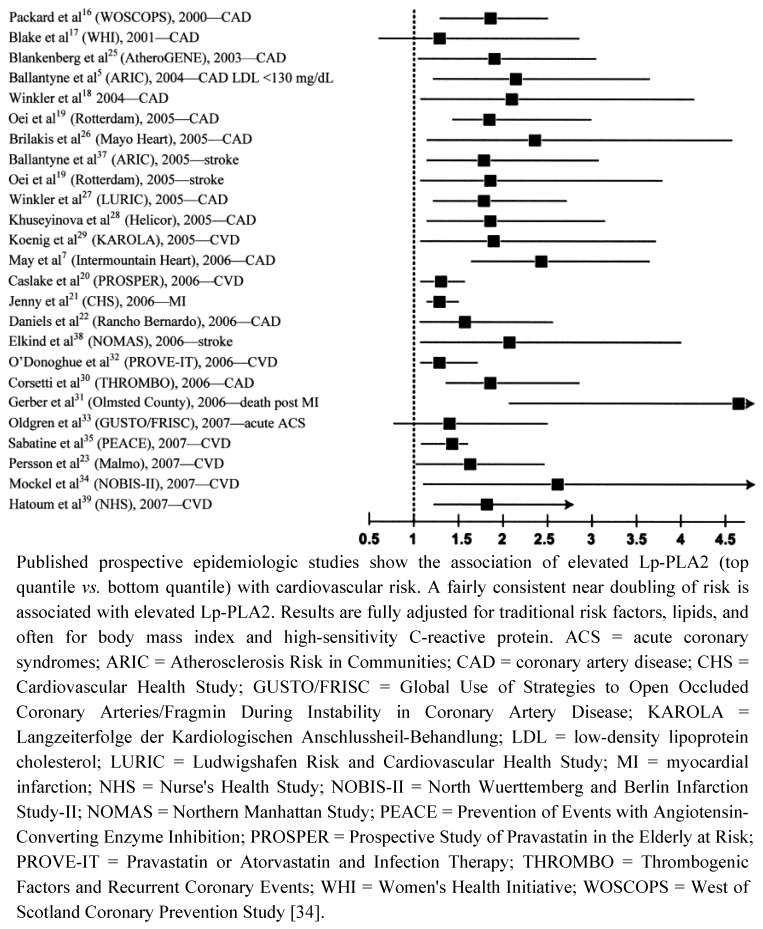
Elevated lipoprotein-associated phospholipase A2 (Lp-PLA2) is consistently associated with a doubling of risk for cardiovascular disease (CVD).

To date, the vast majority of prospective studies analyzing the association between Lp-PLA_2 _ and subsequent cardiovascular (CV) events demonstrated a strong, positive, statistically significant and independent association between increased Lp-PLA_2_ mass or elevated activity and future CV risk, including a wide variety of clinical settings, *i.e.*, apparently healthy men and women, elderly subjects, patients with acute coronary syndrome (ACS), and with stable CHD (Figure 2). Garza *et al.* conducted a meta-analysis summarizing the results of 14 prospective long-term studies with a total of 20,549 participants [31]. They found a significant and independent association between elevated Lp-PLA_2 _concentrations and activity and risk of CVD, resulting in a summary odds ratio (OR) of 1.60 (95%CI, 1.36–1.89) after adjustment for conventional CV risk factors.

### 2.1. Lp-PLA_2_ in Apparently Healthy, Middle-Aged Subjects

The potential association between Lp-PLA_2_ and cardiovascular outcome was first demonstrated in WOSCOPS (West of Scotland Coronary Prevention Study), where 580 hypercholesterolemic middle-aged, initial healthy men, who developed a CHD event over a 4.9-year follow-up (FU), served as cases and were compared to 1,160 matched event-free controls [32]. A one standard deviation (SD) increase in Lp-PLA_2_ concentrations was independently associated with a relative risk (RR) of 1.18 (95%CI 1.05–1.33) for a future CHD event after multivariate adjustment. By contrast, the subsequent Women’s Health Study (WHS), that had included only 123 cases and 123 controls in a nested case-control design, failed to confirm a significant association [33]. 

In the large Atherosclerosis Risk in Communities (ARIC) study, conducted in 608 men and women with incident CHD and 740 controls, and followed for at least six years, after multivariable adjustments, Lp-PLA_2_ was not associated with an increased risk for CHD, except in subjects with LDL cholesterol below the median of 130 mg/dL [35]. In this subgroup, Lp-PLA_2_ significantly and independently predicted CHD (hazard ratio (HR) 2.08; 95%CI 1.20– 3.62), suggesting that it might be a useful marker for identifying patients at risk in those with low and intermediate cardiovascular risk. We had determined Lp-PLA_2_ concentrations in 934 initially healthy, middle-aged men in the MONICA-Augsburg cohort study [36]. Baseline levels of Lp-PLA_2_ were higher in subjects who experienced a coronary event (295 ± 113 *vs*. 263 ±79 ng/mL; p < 0.01), and after multivariate adjustment, including the TC/HDL-C ratio as the strongest lipoprotein variable, a one SD increase in Lp-PLA_2_ was strongly and independently related to a first-ever event (HR 1.23; 95%CI 1.02–1.47). Importantly, like in the ARIC study we evaluated the potential additive value of Lp-PLA_2_ to high-sensitive (hs) CRP in predicting risk. For this purpose, elevated hs CRP was defined according to a recent AHA/CDC consensus document as >3.0 mg/L, and for Lp-PLA_2_ the upper tertile cut-point was used (422 ng/mL in ARIC and 290.8 ng/mL in MONICA). In ARIC, individuals with high Lp-PLA_2_ and high CRP exhibited a threefold increased risk for CHD (HR 2.95; 95%CI 1.47–5.94), whereas in our study the combination of elevated Lp-PLA_2_ and elevated CRP resulted in a HR of 1.93 (95%CI 1.09–3.40) compared with both markers not being increased in the fully adjusted model. Furthermore, in the Rotterdam study, Oei *et al.* [37] measured Lp-PLA_2_ activity in 308 CHD cases and a random sample of 1,820 subjects and followed them for a median of 7.2 years. After controlling for a variety of potential confounders, a one SD increase in Lp-PLA_2_ activity was strongly and independently related to a first-ever CV event (HR 1.20; 95%CI 1.04–1.39), almost identical to findings in the MONICA Augsburg cohort. Kiechl *et al.* [38], in, a population-based survey of 765 men and women aged 40–79 years, the Bruneck study, who were followed over a 10-year period demonstrated in multivariable analysis a HR of Lp-PLA_2_ for subsequent coronary events of 1.4 per one SD change in enzyme activity.

### 2.2. Lp-PLA_2_ in the Elderly Population

Although the predictive value of Lp-PLA_2_ seems to be slightly smaller in the elderly population, it still has been found to be a potent predictor of CVD [34]. The Rancho Bernardo Study [39] enrolled 1,077 initially healthy men and women with a mean age of 72 years, and showed a 60% to 90% increased risk for incident CHD across extreme quartiles of the Lp-PLA_2_ distribution after multivariable adjustment. Data from the Cardiovascular Health Study (CHS), an elderly population without a history of vascular disease at baseline, partially confirmed these results [40]. Only increased Lp-PLA_2_ mass, but not an elevated Lp-PLA_2_ activity was found to be associated with an increased 10-year risk of myocardial infarction (MI) independently of traditional cardiovascular risk factors. Similarly, in the PROSPER (The Prospective Study of Pravastatin in the Elderly at Risk) trial only Lp-PLA_2_ mass was found to be significantly related to future CHD risk, while no association was found for enzyme activity after controlling for various confounders [41]. 

### 2.3. Lp-PLA_2_ in Patients with ACS

Data on the predictive value of Lp-PLA_2_ in the setting of an ACS still remains controversial. While several studies investigating the association between baseline Lp-PLA_2_ activity and -mass and CV events yielded fairly strong associations, like the German NOBIS-II study and data from Olmsted County, Minnesota, the PROVE IT-TIMI 22 trial, the FRISC II trial, and the GUSTO IV ACS study failed to establish baseline levels of Lp-PLA_2_ activity and -mass as an independent risk marker of recurrent CV events [42,43,44,45]. Different time intervals between the index event and blood sampling most likely account for these differences. 

### 2.4. Lp-PLA_2_ in Patients with Stable CHD

By contrast, data regarding the role of Lp-PLA_2_ in patients with stable CHD seems fairly consistent. Brilakis *et al*. [46] were the first to report on Lp-PLA_2_ in patients with pre-existing CHD. They enrolled 466 consecutive patients, who were followed for a median of four years. In multivariable analyses, the RR for a future event for a one SD increase in Lp-PLA_2_ mass was found to be 1.28 (95% CI 1.06–1.54). In the KAROLA (Langzeiterfolge der KARdiOLogischen Anschlussheilbehandlung) study, Lp-PLA_2_ mass and activity were measured on the average 43 days after an acute event in a cohort of 1,051 patients aged 30–70 years with CHD [47]. In multivariable analyses after four years of FU, Lp-PLA_2_ mass was shown to possess prognostic value, whereas Lp-PLA_2_ activity became only borderline significant. Even after adjusting for markers of renal function (*i.e.* cystatin C), and hemodynamic stress (NT-proBNP) there was still an 2-fold increased risk for future CVD events in patients in the upper two tertiles of Lp-PLA_2_ mass compared with the bottom tertile (HR 2.09; 95% CI 1.10 to 3.96). The THROMBO (Thrombogenic Factors and Recurrent Coronary Events) study further confirmed these findings in 766 post-MI patients, who were followed for 26 months [48]. In the large PEACE (Prevention of Events with Angiotensin-Converting Enzyme Inhibition) trial, Lp-PLA_2_ mass was measured in 3,766 patients with documented CHD. After five years of FU elevated Lp-PLA_2_ concentrations predicted adverse CV outcomes [49]. Interestingly, these effects were more pronounced for the prediction of non-fatal events such as revascularization and unstable angina pectoris (UAP). Within the LURIC (Ludwigshafen Risk and Cardiovascular Health) study Lp-PLA_2_ activity predicted risk for cardiac and total mortality over 5.5 years in 2513 patients with angiographically confirmed CHD and 719 without, and added prognostic information in patients with low and medium CRP concentration with regard to 5-year cardiac mortality independently of established risk factors [50]. 

## 3. Clinical Studies

### 3.1. Lp-PLA_2_ and Endothelial Dysfunction

Recent studies have demonstrated that Lp-PLA_2_ is associated with endothelial dysfunction and early atherosclerosis. Yang *et al.* [51] recruited 172 patients without significant CAD in whom coronary endothelial function was assessed in response to intracoronary acetylcholine. The OR for presence of coronary endothelial dysfunction in patients with Lp-PLA_2_ in the highest tertile was 3.3 (95% CI, 1.6 to 6.6). In another study, coronary angiography, blood flow, flow reserve, endothelial function, and intravascular ultrasound with volumetric analysis were performed in 15 patients with mild coronary atherosclerosis and in 15 control subjects [52]. Plasma samples were collected simultaneously from the left main coronary artery and coronary sinus for measurement of Lp-PLA_2_, Lyso-PC, and CRP. While CRP was not significantly different between the groups, net production of Lp-PLA2 and Lyso-PC in the coronary circulation was higher in patients compared with control subjects, and correlated with coronary endothelial dysfunction.

### 3.2. Lp-PLA_2_ as A Target for Pharmacologic Intervention

Since there is increasing evidence for a pivotal role of inflammation in atherothrombosis, and the main downstream product of Lp-PLA2, Lyso-PC represents a potent pro-inflammatory molecule, Lp-PLA2 may not only be a predictor of CVD risk as discussed above but may also become a therapeutic target.

**Table 1:** Clinical trials evaluating darapladib.

Author [Ref.]	Phase	Mass or activity	Cohort	*N*	Treatment	Primary Endpoint	Significant effects
Johnson *et al.* [56]	II	Lp-PLA_2_ activity	Patients before elective endarterectomy	59	14 days	Lp-PLA_2_ activity	80% reduction in enzyme activity
Mohler *et al.* [57]	II	Lp-PLA_2_ activity	CHD and CHD-risk equivalent patients receiving atorvastatin	959	12 weeks	Lp-PLA_2_ activity and biomarkers	43-66% reduction in enzyme activity, 13% reduction of IL-6, and CRP (p = 0.028 and p = 0.15)
Serruys *et al.* [58]	II	Lp-PLA_2_ activity	Patients with angiographically documented CHD	330	12 months	Coronary atheroma plaque cap deformability; CRP	59% reduction in enzyme activity, halt of necrotic core expansion (-5.2 mm^3^; p = 0.012)

This Table summarizes results from three phase II clinical trials. Modified with friendly permission after [53].

Darapladib, a selective inhibitor of Lp-PLA_2 _ is a small molecule which was developed in 2003 by GlaxoSmithKline (GSK) [54]. Darapladib convincingly demonstrated beneficial effects in a diabetic/hypercholesterolemic pig model. These animals were randomly assigned either to a control group or a treatment group receiving 10 mg/kg darapladib per day [55]. After 24 weeks, Lp-PLA_2_ activity in plasma was reduced by 89% in the treatment group (p < 0.00001 *vs.* placebo), and coronary gene expression analyses revealed a substantial reduction of the expression of 24 genes associated with macrophage and T-cell function. Furthermore, the study indicated that selective Lp-PLA_2_ inhibition may promote lesion stabilization. The median plaque area in the left anterior descending coronary artery was significantly reduced from 0.222 mm^2^ to 0.086 mm^2^ (p < 0.05), and seven out of 17 control pigs showed a fibrous or thin fibrous cap atheroma compared to only two out of twenty in the darapladib group (41% *vs.* 10%; p = 0.05). In addition, the necrotic area from the arterial section with the greatest plaque area was significantly reduced in the treatment group (0.87 ± 0.33 mm^2^ *vs.* 0.03 ± 0.003 mm^2^; p = 0.015 *vs.* placebo). To date darapladib has demonstrated efficacy in three multicenter, randomized, double-blind, placebo-controlled trials [56,57,58] (Table 1).

In an early phase II clinical trial [56], the administration of two different doses of darapladib for 14 days before elective carotid endarterectomy in 59 patients resulted in a significant systemic inhibition of Lp-PLA_2_ plasma activity by 80%, and a significantly reduced local Lp-PLA_2_ activity in atherosclerotic plaque. Furthermore, IL-18 levels and activity of the pro-apoptotic caspase-3 and caspase-8 were attenuated compared to placebo.

Mohler *et al.* [57] tested the effects of darapladib (40, 80 and 160 mg, respectively) on Lp-PLA_2_ activity and on biomarkers of CV risk in 959 CHD and CHD-risk equivalent patients receiving aggressive lipid-lowering therapy (atorvastatin 20 or 80 mg per day) (NCT00269048). After 12 weeks of therapy, darapladib inhibited Lp-PLA_2_ activity in a dose-dependent manner by approximately 43%, 55%, and 66% compared with placebo (p < 0.001 *vs.* placebo). Furthermore, IL-6 and CRP displayed a strong decrease in the high-dose treatment group (12.6% and 13.0% decrease, respectively; p = 0.028 and p = 0.15 *vs.* placebo, respectively). In contrast, levels of total cholesterol, LDL- and HDL cholesterol were not modified as compared with placebo. Of special note, no major safety concerns were noted after 12 weeks of treatment. 

The international, multicenter, randomized, double-blind, placebo-controlled IBIS-2 (Integrated Biomarker and Imaging Study-2, [58]) tested the effect of 12 months of treatment with darapladib 160 mg daily in 330 patients with angiographically documented CHD. After 12 months, treatment with darapladib resulted in significantly reduced Lp-PLA_2_ activity levels (59% inhibition, p < 0.001 *vs.* placebo), while the primary endpoints of the study -atheroma deformability measured by palpography (p = 0.22 *vs.* placebo) and plasma CRP lowering (p = 0.35 *vs.* placebo) were not met. Also, HDL- and LDL cholesterol were unaffected by treatment. However, despite adherence to a high level of standard-of-care treatment, in the placebo-treated group the necrotic core volume increased significantly (4.5 ± 17.9 mm^3^; p = 0.009), whereas darapladib halted this increase (-0.5 ± 13.9 mm^3^; 0.71) in the intervention group, resulting in a significant treatment difference of –5.2 mm^3^ (p = 0.012). These intra-plaque compositional changes occurred without a significant treatment difference in total plaque volume or calcification (p = 0.95). Regarding clinical safety, higher systolic casual blood pressure in the darapladib group (3.0 mmHg, 95%CI 0.3-5.7; p = 0.031) was not consistent with results of the prior clinical study, and additional comparison of the intra-arterial blood pressure also revealed no differences between groups. Treatment-emergent adverse effects did not significantly differ from placebo. 

In December 2008 GSK initiated the STABILITY trial (NCT00799903), a phase III, randomized, double-blind, placebo-controlled, parallel-assigned, multicenter clinical outcome trial in 15,500 patients with chronic CHD [59]. In this event driven trial, darapladib is assessed on top of standard CHD pharmacotherapy, including high dose statins. The primary outcome measure is determined as time to first occurrence of any component of the composite of major adverse cardiovascular events, consisting of cardiovascular death, non-fatal myocardial infarction and non-fatal stroke. Final results of the study will be available by 2012, or until around 1,500 major adverse CV events have occurred. This trial will test the inflammation hypothesis and will provide more insight into whether or not targeting inflammation carries clinical benefit for the high risk patient with CVD. In spring 2010 another randomized controlled clinical trial of similar size, SOLID, will be launched in post-ACS patients. 

## 4. Disclosures

M. Karakas declares that he has no financial or personal relations to other parties whose interests could have affected the content of this article in any way, either positively or negatively. W. Koenig declares that he is a member of the Steering Committee of the STABILITY Trial, and has received honoraria for lectures from GSK.
